# Action observation: the less-explored part of higher-order vision

**DOI:** 10.1038/srep36742

**Published:** 2016-11-18

**Authors:** Artem Platonov, Guy A. Orban

**Affiliations:** 1 Department of Neuroscience, University of Parma, Parma, Italy

## Abstract

Little is presently known about action observation, an important perceptual component of high-level vision. To investigate this aspect of perception, we introduce a two-alternative forced-choice task for observed manipulative actions while varying duration or signal strength by noise injection. We show that accuracy and reaction time in this task can be modeled by a diffusion process for different pairs of action exemplars. Furthermore, discrimination of observed actions is largely viewpoint-independent, cannot be reduced to judgments about the basic components of action: shape and local motion, and requires a minimum duration of about 150–200 ms. These results confirm that action observation is a distinct high-level aspect of visual perception based on temporal integration of visual input generated by moving body parts. This temporal integration distinguishes it from object or scene perception, which require only very brief presentations and are viewpoint-dependent. The applicability of a diffusion model suggests that these aspects of high-level vision differ mainly at the level of the sensory neurons feeding the decision processes.

Action observation refers to the process of visually assessing the goal of an action performed by conspecifics, as well as how the movements of the effectors allow achieving that goal. Although the visual processing of others’ actions has been touched upon in the neurophysiological studies of Perrett and coworkers^1^ and theoretical studies^2^,^3^, few psychophysical studies have undertaken this aspect of higher-order vision. Most behavioral studies devoted to action observation have used complex sequential analysis tasks requiring multiple high level cognitive processes^4^,^5^,^6^, combining action naming, deception, detection and prediction tasks^7^, or interactions with action execution^8^. Observed actions have frequently been reduced to point light displays^9^ or even static pictures^10^. Very few studies^11^ have made use of discrimination tasks which have proved so productive in studying other aspects of vision^12^,^13^. As a consequence, relatively little is understood about the perception of others’ actions (observed action perception, OAP) in contrast to the wealth of information published regarding object and scene perception^14^,^15^,^16^.

To address this void, we have employed two types of two-alternative, forced-choice discrimination tasks based upon observed actions: one in which perception is degraded by injecting dynamic noise into the action videos (experiments 1–5), and a second task in which the visibility of the observed actions is limited in duration by backward masking with dynamic noise (experiment 6). The stimuli used are natural actions, i.e. video recordings of humans performing actions, rather than reduced stimuli such as point-light displays^17^,^18^,^19^,^20^ or stick figures^21^. The noisy discrimination task is introduced to demonstrate that the discrimination of observed actions can be modeled by a proportion-rate diffusion model (experiments 1–2), as has been shown for many other discrimination tasks^22^. As in earlier studies of the discrimination of dynamic stimuli^23^, two versions of the noisy observed actions discrimination task will be used: one in which subjects respond at the end of the video and one in which subjects respond as soon as ready. This task will be used to assess the viewpoint-dependence of action observation (experiment 3) and the nature of the visual information used in this visual process by comparing performance for actions to that for their static shape or dynamic local motion components (experiments 4–5). In the present study, the observed actions all belong to the class of manipulative actions, i.e. actions intended to displace or modify an object, the neuronal substrate of which has been investigated in several recent imaging studies^24^,^25^.

## Materials and Methods

The aim of experiments 1 and 2 was to establish that the proportion-rate diffusion model applies to perceptual discrimination between two observed manipulative actions, either for the pair rolling and rotation (experiment 1), or for dragging and grasping (experiment 2). The goal of experiment 3 was to examine the influence of the observer’s viewpoint on his/her ability to discriminate between actions. Experiments 4 and 5 tested for alternative interpretations of the first 3 experiments in terms of lower-level mechanisms that might account for the results. Finally, in experiment 6, we determined the dependence of action perception on action duration, as an alternative to noise injection for reducing visual action signals.

### Subjects

Thirty two healthy human subjects with normal or corrected to normal visual acuity participated. Nine subjects took part in experiment 1 (S1–S9), 4 in experiment 2 (S2, S3, S6 and S9), 12 in experiment 3 (S10–S21), 4 in experiment 4 (S1–S3 and S22), 4 in experiment 5 (S2, S3, S9 and S22) and 10 in experiment 6 (S23–S32). All subjects were naïve as to the purpose of the experiments and gave informed consent for participation. Experiments were carried out according to the national and European guidelines for testing human subjects, and all experimental protocols were approved by the Ethical committee of the Province Parma.

### Setup

Subjects were seated 72 cm from a liquid crystal display (Samsung, T27A950, resolution 1920 × 1080 pixels, 50 Hz refresh rate) in an otherwise dark room with their heads supported by a forehead rest and a chin cup. The visual stimuli were generated by a personal computer equipped with an open GL graphics card using the Psychophysics Toolbox extensions^26^,^27^ for Matlab (The Math Works, Inc.). We used a precision Minolta Luminance Meter LS-100 to calibrate the display, setting its mean brightness to 50cd/m^2^ for all experimental conditions.

Subjects were instructed to fixate a small target in the center of the screen. Eye movements were recorded using a noninvasive monitor-mounted infrared video system (Tobii Version X2-60) sampling the positions of both eyes at 60 Hz under the control of the Tobii Toolbox extensions of Matlab Version 1.1^28^. Trials were rejected if the percentage of blinks exceeded 5%. For every successful trial, we analyzed the position of the less noisy of the two eye recordings. Fixation performance was similar in all experiments, and the standard deviation of eye position over the six experiments averaged 1.11° ± 0.45 horizontally and 0.85° ± 0.40 vertically (Supplementary Table 1).

### Visual stimuli

In all experiments, the *experimental* stimuli consisted of video clips (17^o^ × 13^o^, 50 Hz) showing a human actor manipulating an object. Since the main interest of the study was observed action perception, only allocentric viewpoints were tested. Observers had to discriminate between the actions viewed either from the side, or in half of the trials of experiment 3, from a frontal viewpoint. Because the action was occluded by the actor’s hand when seen from his/her right in one of the two tested action pairs (rolling and rotation), only one lateral viewpoint was tested (the actors’ left and observers’ right sides, Supplementary Figure 1). Video clips lasted 2.6 s in experiments 1–5, and 1.5 s in experiment 6. The actor manipulated the object using his/her right hand which was positioned either above (experiments 1–5 and half of the trials in experiment 6) or on (experiment 2 and half of the trials in experiment 6) a table. The hand motion started 20–60 ms from the beginning of the movie in experiments 1–5, and at 100 ms in experiment 6. Video edges were blurred with an elliptical mask (14^o^ × 10^o^), leaving the action and the hand and face of the actor together with the background unchanged but gradually blurring into the black background around the edges. Each movie contained a fixation cross located at the same position on the screen for all the videos presented.

In experiments 1, 3, 4, and in half the trials of experiment 5 we tested discrimination between rolling and rotating (action pair 1, Supplementary Figure 1A) using either a small sphere or cube (0.2^o^). When rolling an object, fingers and thumb work in opposite directions, whereas, in rotation, fingers and thumb must work together moving the object around a vertical axis. The hand extended 1.13^o^ horizontally. In experiment 2, in half the trials of experiment 5, and in experiment 6, we tested discrimination between dragging and grasping (action pair 2, Supplementary Figure 1B) taken from Ferri *et al*.^25^. A blue ball (0.3^o^) and a slightly smaller red cube (0.25^o^) were manipulated in these actions involving wrist and fingers (Table 1). The hand measured 2.05^o^ horizontally.

In experiments 1–5, we created 40 versions of each action exemplar by combining 2 actors ( male, female) × 2 objects (ball, cube) × 5 fronto-parallel positions (central plus four positions at 2.5° eccentricity along the diagonals) × 2 sizes (standard, 20% larger). Each version was then degraded using different quantities of dynamic noise. To do this, for each pixel of every movie frame, we randomly chose a corresponding pixel located within a predefined distance (PD). These two pixels were interchanged with a probability (P_s_) set by the experimenter, thus creating a signal level (SL) defined as 100-P_s_. This manipulation was repeated for each frame of a given movie. Table 1 gives an overview of all PD and SL values used to test action pairs in experiments 1–5. In experiment 6, we created 32 versions of each action exemplar by combining 2 actors (male, female) × 2 objects (ball, cube) × 2 hand postures (open palm, fist) × 2 hand positions (on the table, above the table) × 2 sizes (standard, 20% larger). Each action movie was presented at 100% SL for 100, 260, 400, 560, and 700 ms after its onset, and was then replaced by the dynamic mask, created by setting SL for the remaining part of the video clip to 0%.

In experiments 4 and 5, two types of *control* stimuli were presented: dynamic and static control stimuli were tested in experiment 4, while in experiment 5 only static stimuli were used. The dynamic control stimuli were generated for each version of the video clips following the procedure described in Ferri *et al*.^25^. Local motion vectors were computed for each pixel on a frame-by-frame basis^29^ and used to animate an anisotropic noise pattern. Subsequently, this dynamic pattern was temporally scrambled by dividing each video frame into 65 squares, whose size gradually increased from the center of the action (central square = 0.1^o^) to the periphery (outmost square = 2^o^), and randomizing the starting frame for each square. Finally, we replaced the optic flow in each square with a uniform translation having the speed and direction of the mean optic flow (average over the square). These manipulations eliminated any perception of a moving human upper limb, but held the local motion, mean contrast and brightness within each square the same as in the original videos.

The static control stimuli were single frames taken from the videos and presented for 2.6 seconds. These were selected differently in experiment 4 and 5. In experiment 4, we defined 40 time points uniformly distributed across the duration of the video clip and pseudo randomly selected the corresponding frame from the 40 videos available per action (Supplementary Figure 2). This ensured that the static frames encompassed all 40 versions as well as covering the entire duration of the action videos. In Experiment 5 a static frame from the beginning (100 ms after video onset), the middle, or the end of the action video (100 ms before end) was selected for each of 40 versions of each action exemplar, a procedure similar to earlier fMRI studies^25^.

### Task

In all experiments, we used a two-alternative forced-choice (2AFC) action discrimination task in which subjects viewed a single video clip and indicated their choice of two possible actions by pressing one of two buttons with the right hand. Subjects had to fixate upon a cross near the center of the screen for the duration of each trial. During the 2 s inter-trial interval only the fixation cross was visible. Subjects gave a response either as soon as they could answer (experiments 1–3) or within 1 s of the end of the movie (initial training in experiment 3, experiment 4, 5 and 6).

### Training and test procedures

Before participating in experiments 1–5, all observers received equal training in discriminating between the rolling and rotating actions (see Supplementary Information). Each experimental session included one or two blocks of testing. To ensure that the subjects remembered the task in experiments 1–5, each session was preceded by a familiarization block (30 no-noise trials). The results of the familiarization blocks were included in the data analysis of experiments 1 (S6–S9), 2 and 3 as a 100%-signal data point (see *Results*). In all experiments subjects had to discriminate between 2 actions (Table 1). In addition, in experiments 4 and 5, subjects performed the same task while being presented with static and dynamic control stimuli.

Action stimuli were presented in random order in all experiments. In experiments 1–3, 480 (40 × 2 × 6) trials were split into 2 blocks of 240 trials each, tested in a single session. In experiment 4, 1440 (40 × 2 × 6 × 3) movie clips were split into 4 blocks of 360 trials each and tested in four sessions. In experiment 5, 800 (40 × 4 × 5) trials were split into 4 blocks of 200 trials each and tested in four sessions. In experiment 6, 320 (32 × 2 × 5) tested in one session.

### Data analysis

The data collected in experiments 1–3 were fitted with the proportional-rate diffusion model (following ref. 30), in which bound and drift rate were normalized by the diffusion coefficient reducing the number of free parameters to 3: the normalized bound (*A’*), the mean residual time (*t*_*R*_) and the mean sensitivity (*k*).

A significant advantage of a diffusion model over the models, which focus on either accuracy or response time measures, is that it optimizes the usage of information obtained in the experiment by assessing both accuracy and response time using a common metric^31^,^32^. The model predicts that the psychometric function for accuracy *P*_*C*_(*x*) is a logistic function of the percentage of signal *x*:


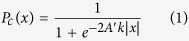


The model prediction for chronometric function of the mean response time *t*_*T*_(*x*) is


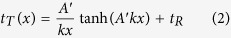


in which percent signal enters the function as both a 1/*x* term and as an argument for the hyperbolic tangent function.

The free parameters were fit using the maximum likelihood method. We also calculated 75% accuracy thresholds (halfway between chance and perfect performance) and estimated the halfway response time threshold midway between the extreme values of the response time curve. We also calculated the threshold ratio: halfway time thresholds/75%-accuracy threshold, the value of which has typically been reported to be close to 3.5 for the diffusion model^30^.

The diffusion model is applicable to versions of the task in which subjects respond as soon as ready, i.e. Experiments 1 and 2 and sessions following the initial training of Experiment 3. Since subjects responded at the end of each trial in the remaining session and experiments, the diffusion model did not apply. Hence, data were fitted with a logistic regression, whereby the probability of a rotation-choice (P_Rot_) or of dragging-choice is given by


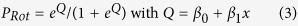


where *x* is percent signal, using the convention that positive values indicate rotation and negative values rolling in Experiments 3, 4 and 5 (2 subjects) or dragging and grasping in Experiments 5 (remaining 2 subjects) and 6 respectively, and the β_i_ variables are free parameters fitted using the maximum likelihood method^33^. As shown by Palmer *et al*.^30^, such logistic regression functions produce a reliable description of choice behavior in two-alternative forced-choice visual motion discrimination tasks.

The Anderson-Darling goodness-of-fit hypothesis test was applied to the data. Since this test did not reject the hypothesis proposing normal distribution of the calculated thresholds, post hoc comparisons of the thresholds were carried out with analysis of variance (ANOVA) and Student’s *t* tests.

## Results

### Experiment 1

**Figure 1:**
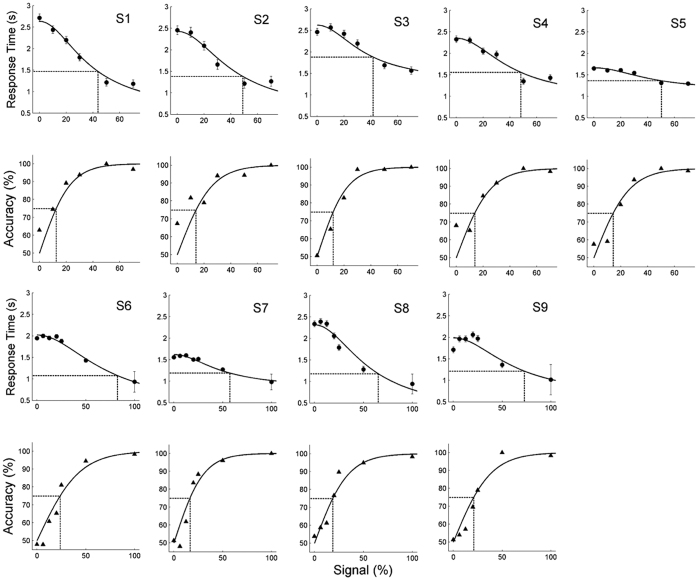
Experiment 1. Response time (circles, upper rows) and accuracy (triangles, lower rows) plotted as a function of signal strength for 2AFC discrimination of rotating-rolling by subjects S1–S9. The proportional-rate diffusion model provided a close fit (solid lines) to the data in all subjects. Dashed lines indicate halfway response time and 75% accuracy thresholds (see Table 2). Error bars indicate ±1 SEM.

In experiment 1, subjects discriminated between rolling and rotating. First, we tested the validity of the assumption made by the model, that the bounds (*A’*) should be symmetrical relative to the starting point, meaning that subjects are not biased towards one of the two alternatives. The estimated response bias (c) was calculated after Macmillan & Creelman^34^ and was negligible (c < 10^−14^) in all subjects (N = 9) and for all signal strength conditions. Next, we combined responses to the two actions to express performance as a single variable, accuracy, ranging from 50 to 100%, as shown in Fig. 1. Both accuracy (triangles) and response times (circles) of all subjects were closely fitted (Fig. 1) by the proportion-rate diffusion model (solid lines), the three free-parameter values of which are listed in Table 2. Note that the values of *k* varied across subjects in the same direction as *t*_*R*_: slower accumulation of evidence leaves less residual time (Table 2). The parameter values, although larger and more variable across subjects than those reported earlier for a motion-direction discrimination task^30^, are within similar ranges. Finally, from the fit of the model to the data, we calculated the halfway response time and 75% accuracy thresholds. These were closely coupled since the threshold ratios were near 3.5 in all subjects (Table 2). The diffusion model described the data well, as indicated by the robust correlations (Supplementary Figure 3) between predicted and measured accuracy (r = 0.98; t-test, p < 0.01) and response time (r = 0.94; t-test, p < 0.01). Experiment 1 thus indicates that the diffusion model indeed applies to the observed action discrimination in the 2AFC task.

### Experiment 2

**Figure 2:**
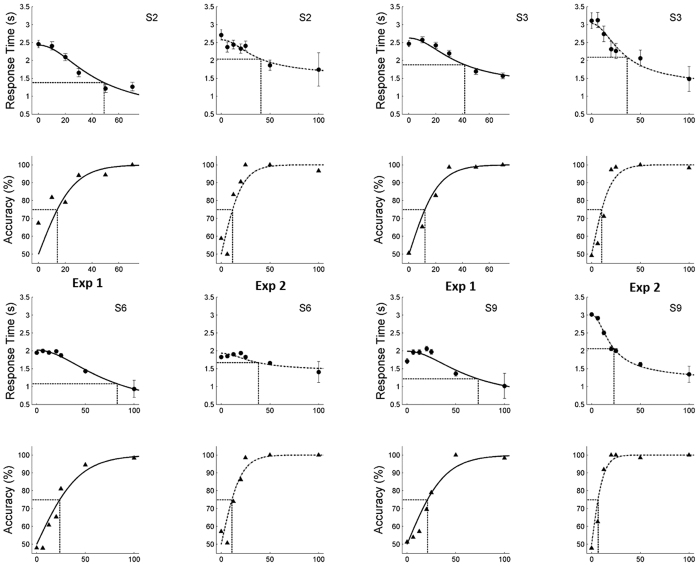
Experiment 2. Response time (circles, upper rows) and accuracy (triangles, lower rows) plotted as a function of signal strength for discrimination of grasping-dragging by subjects S2, S3, S6, S9. To facilitate the comparison between the 2 action pairs, results from the same subjects obtained in experiment 1 are also plotted. Neither the halfway response times nor 75% accuracy threshold, calculated from the proportional-rate diffusion model fit (lines) differed significantly between two action pairs tested. Same conventions as Fig. 1.

In experiment 2, we tested the generality of the results obtained in experiment 1 by presenting subjects with another pair of manipulative hand actions (dragging and grasping). Figure 2 compares the response times (circles) and accuracies (triangles) obtained for rolling/rotation (experiment 1) and dragging/grasping (experiment 2) action pairs. The proportion-rate diffusion model provided a close fit (solid lines) to the data of experiment 2, as it did in Experiment 1 (Table 2). This is further indicated (Supplementary Figure 4) by the strong correlations between predicted and measured accuracy (r = 0.93; t-test, p < 0.01) and response time (r = 0.94; t-test, p < 0.01).

While thresholds were lower in experiment 2 than experiment 1, neither the 75% accuracy thresholds (paired t-test, t (6) = 2.12, p > 0.07) nor the halfway response time thresholds (paired t-test, t (6) = 2.13, p > 0.07) differed statistically between the experiments. Note that averaging the local motion vectors (extracted with the same algorithm as used to generate dynamic controls, see methods) within the actions over time showed that dragging and grasping contain more than 3 times the number of local motion vectors than in rolling and rotation, and that their average amplitude is over 3.5 times that of rolling and rotation. This can be taken as an indication that the dynamic changes in shape that characterize the actions are greater for the dragging/grasping than for the rolling/rotating pair, and that one may therefore expect a better discrimination performance. One possible explanation for the lack of significant differences might be that all subjects were initially trained in discrimination between rolling and rotation actions (see Training procedure), facilitating later processing of these previously viewed stimuli, even after a considerable period of time.

### Experiment 3

So far, we have shown that observed action discrimination can be fit with the diffusion proportion-rate model for two pairs of manipulative action exemplars observed from the side. In Experiment 3, we compared observed action discrimination at two viewpoints: lateral and frontal. To that end, we trained a new group of subjects to discriminate between rolling and rotation actions viewed from either the front or the right side (of the observer, see methods), and then tested their performance before and after switching the viewpoint. Thus, subjects who learned discriminating between the two actions seen from the right side were presented with the same actions viewed from the front, and vice versa. It is important to note that the two viewpoints did not provide equal amounts of information about the actions, since the frontal viewpoint displayed mainly the fingertips, while in the lateral view most of the fingers and thumb were visible.

Table 3 shows the calculated 75%-accuracy thresholds obtained in the group 1 (S10–S15) subjects trained for the frontal viewpoint and then tested for the lateral viewpoint, and group 2 (S16–S21) subjects, first trained for the lateral viewpoint and then tested for the frontal viewpoint. The data represent subjects’ performance in the initial (IT), middle (MT) and final (FT) training sessions and the session after the viewpoint switch (VS). Thresholds were calculated using logistic regression for the IT session and the diffusion model for MT, FT and VS sessions. Table 4 shows the halfway response time thresholds for the same groups of subjects in sessions MT, FT and VS. The parameters of the diffusion model for these last three sessions are shown in Supplementary Table 3.

A two-way ANOVA applied to the accuracy thresholds of training sessions (Table 3) yielded a main effect of the viewpoint on (F_1, 35_ = 13.7, p < 0.01) indicating larger thresholds for the subjects using the frontal viewpoint during training. Since the threshold ratio is very close to 3.5 in all sessions (Supplementary Table 4), the response time threshods were larger in group 1 subjects (Table 4). One possible explanation may lie in the difference in the amount of information provided by the 2 viewpoints. While the lateral viewpoint reveals the motion of fingers and thumb during manipulation, the action information in the frontal viewpoint is represented mainly by the motion of fingertips. Indeed, the amount of dynamic shape changes (using the local motion vectors as proxy, see above) in the two viewpoints differs by more than a factor 2. Thus, the explanation for the initial viewpoint effect might be similar to that for the difference in thresholds between experiments 1 and 2. The main effect of sessions, however, indicates also that thresholds decrease with time, indicating a signifcant training effect, which again applies also to the time data, given the threshold ratios (Supplementary Table 4). After the switch, the thresholds are extremely similar and differences between accuracy thresholds are not signifcant (t(10) = 0.09, p > 0.93). The same is true for the time thresholds (Table 4). Whether the absence of viewpoint effect in the VS session simply results from training away the discrepancy in visual action information provided by the viewpoints or whether it also reflects the familarisation with the action from the other viewpoint requires further study; However the fact that thresholds in group 2 increase only modestly, despite the reduction in visual action information, suggests that the latter factor does contribute. This notion is also supported by the comparison of FT in group 1 with VS in group 2, which both correspond to the frontal viewpoint.

The diffusion model described the data from MT, FT and VS sessions well. This is indicated by the fit of the model to the individual data (Supplementary Figures 5 and 6). It is also corroborated by the correlations between predicted and measured accuracy and response time (Supplementary Figures 7–9) in MT (group 1: r = 0.94; t-test, p < 0.01 and r = 0.91; t-test, p < 0.01, respectively; group 2: r = 0.93; t-test, p < 0.01 and r = 0.96; t-test, p < 0.01, respectively), FT (group 1: r = 0.91; t-test, p < 0.01 and r = 0.89; t-test, p < 0.01, respectively; group 2: r = 0.93; t-test, p < 0.01 and r = 0.88; t-test, p < 0.01, respectively), and VS (group 1: r = 0.91; t-test, p < 0.01 and r = 0.90; t-test, p < 0.01, respectively; group 2: r = 0.94; t-test, p < 0.01 and r = 0.92; t-test, p < 0.01, respectively).’

### Experiment 4

**Figure 3:**
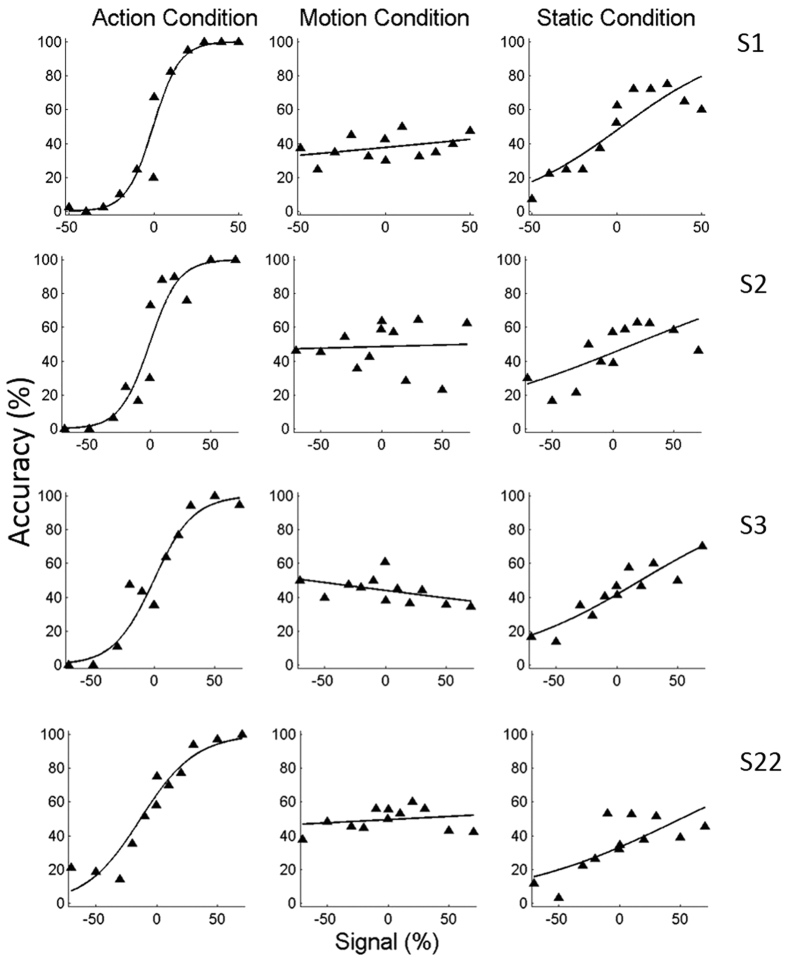
Experiment 4. Accuracy in the manipulative action discrimination task for subjects S1–S3 and S22 in the action, static and dynamic conditions. Positive and negative signal values represent rotation and rolling actions, respectively. A logistic regression fit to the data is superimposed.

This experiment tested whether observed action discrimination is distinct from the perception of its components: static shape and local motion. Figure 3 displays observers’ performance by plotting the percentage of rotation choices (triangles) as a function of signal strength. Positive and negative signal values represent rotation and rolling actions, respectively. The 75% action discrimination thresholds in the action condition, as inferred from logistic regression fits to the data (solid curve), ranged from 8.65- to 24.7% signal level (Supplementary Table 4). Moreover, neither static nor motion components alone could account for these results. Thresholds were much higher in the static condition (64.3 SL ± 20.7) than in the action condition (16.1 SL ± 6.86; paired t-test, t (3) = 4.82, p < 0.02). Extrapolating to 100% signal, subjects would reach only 70 to 80% correct in the static conditions. In the motion condition, the performance remained so low at high SL that no threshold could be calculated. Thus, these results indicate that action discrimination is distinct from shape or motion discrimination.

### Experiment 5

In the previous experiment, the static frame presentation allowed some discrimination to be made between the two action exemplars. However, in the static condition, we tested images taken from many time points during the video and from all versions of the action exemplar. If static frames within an observed action video contain different amounts of information about the action, some frames may be more informative than others. In this case, it might be more efficient to show frames from the same time point in different versions of the actions. Hence, in experiment 5, we tested only 3 static frames taken from either the beginning, middle or the end of the movies, as is routinely done in action observation fMRI studies^25^.

**Figure 4:**
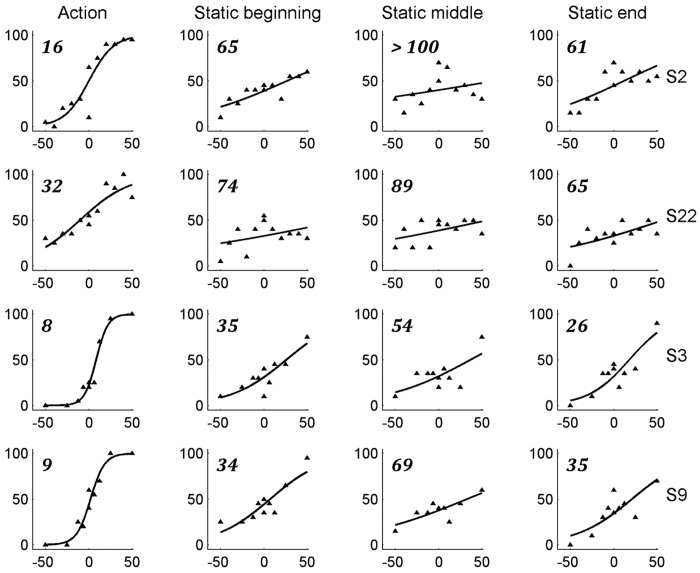
Experiment 5. Accuracy in discriminating between rotation and rolling (S2 and S22) and grasping and dragging (S3 and S9) plotted for the action and static stimuli depicting beginning, middle and end time points. Positive and negative signal values represent rotation and rolling, and dragging and grasping actions, respectively. A logistic regression fit to the data is superimposed. 75% accuracy thresholds (in SL) are indicated by numbers in italic at the top left of the plots.

Figure 4 displays observers’ performance, following the same conventions as Fig. 3, in the action condition and in the static conditions for frames taken from the beginning, middle and the end of the action movie. Two-way ANOVA of the static conditions yielded a significant main effect of the time-position on the observers’ accuracy thresholds (F_1, 11_ = 52.0, p < 0.01). More specifically, a static image taken from the middle of an action evoked much higher thresholds than frames taken from the other 2 time positions: the beginning (paired t-test, t (3) = 6.08, p < 0.05, Bonferroni corrected) or end (paired t-test, t (3) = 6.08, p < 0.01, Bonferroni corrected). Although the thresholds for the 2 action pairs differed significantly (main effect of the action pair, F_1, 11_ = 72.0, p < 0.01), the differences in thresholds at the 3 time positions did not depend on which action pair was tested (action pair × time position interaction F_1,11_ = 0.001, p > 0.95). In addition, there was no significant difference between the images taken from the beginning and the end time points (paired t-test, t (3) = 2.23, p > 0.11). Nonetheless, thresholds in the action condition were less than half the thresholds for the best static conditions, whether these frames were from the beginning of the video (paired t-test, t (3) = 6.08, p < 0.02, Bonferroni corrected) or the end (paired t-test, t (3) = 5.24, p < 0.03, Bonferroni corrected). Again, extrapolating to 100% signal generally yielded performances below 100% correct for most combinations of time-point and action-pair. These results further support the notion that action discrimination cannot be reduced to static shape perception.

### Experiment 6

**Figure 5:**
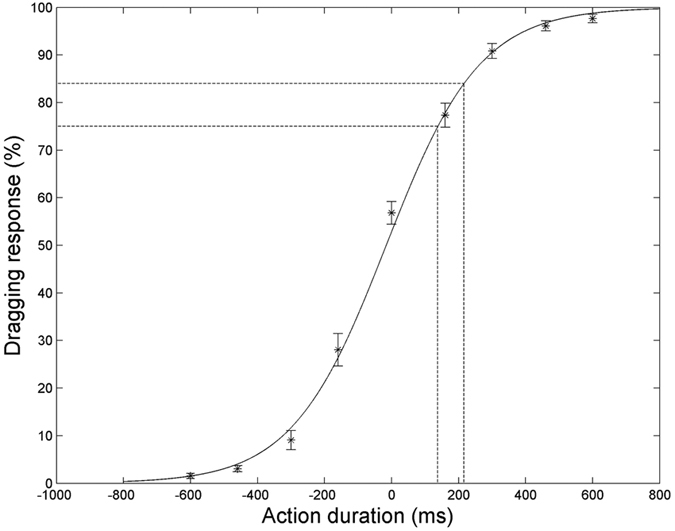
Experiment 6. Average (n = 10) % dragging responses as a function of duration of the observed action: positive and negative durations represent observed dragging and grasping respectively. The logistic regression fit to the data is again superimposed. Vertical bars indicate SEM. 75% and 84% thresholds (dashed lines) averaged 138 ms and 216 ms respectively.

In contrast to the previous experiments in which the video stimuli were degraded by injection of various amounts of dynamic noise, visibility, in experiment 6, was manipulated by restricting the time during which actions could be observed. The action videos always started the same way, but were replaced by dynamic noise at variable times after onset. This manipulation had a strong effect on the accuracy of discrimination especially when action presentation lasted less than 150 ms (Fig. 5). The 75% and 84% thresholds equaled 138 ms and 216 ms from onset of action observation, corresponding to 7 or 11 frames of the video.

## Discussion

### Observed action discrimination modelled by diffusion model

Our results show that performance in the 2AFC task for observed actions can be closely modelled by a proportion-rate diffusion model. This is demonstrated by 1) the close fit of the model to the experimental data (Figs 1 and 2, Supplementary Figures 5 and 6), 2) the correlations between predicted and observed accuracy and reaction times (Supplementary Figures 3,4,7–9) and 3) the ratio of halfway response time and accuracy thresholds for different action-pairs, which is extremely close to 3.5, the value typical for a diffusion process, (Table 2, Supplementary Table 4). Typical response times for human observers in motion-direction discrimination at 100% signal level in a 2AFC task was on the order of 300–400 ms^30^, much shorter than the values obtained here for observed-action discrimination. It could be argued that the longer latencies reported in our study are problematic for modeling action observation by a diffusion process, since it is has been suggested that the diffusion model can be used to describe only relatively fast two-choice decision tasks (mean response times less than 1000 to 1500 ms^35^). However, there is no empirical evidence supporting this proposition^36^. This arbitrarily chosen time-constraint, which significantly restricts the scope of diffusion model applications, can easily be overcome by carefully assessing the model fit (see Supplementary Figures 3,4,6–9). In our study, the mean response time, in some cases, exceeded 3000 ms (experiment 2, S4 and S10), yet the diffusion model closely fitted perceptual decisions concerning observed actions in all cases (Table 2, Supplementary Figure 4).

Thus, the diffusion model applies to noisy observed action discrimination, in agreement with the review by Heekeren *et al*.^22^, indicating a broad applicability of this model to higher-order visual, somatosensory and auditory discrimination tasks. The applicability of the model has two important implications. First, the number of cognitive processes involved beyond sensory processing is indeed limited, involving only a decision stage, contrasting our task with the behavioral tasks used to probe action observation in previous studies. Second, the proportion-rate diffusion model implies a two stage process in which the outputs of neurons selective for the stimuli provide evidence, accumulated by a second stage, where a decision is reached once the accumulated noisy evidence reaches a criterion bound. This interpretation is supported by studies of low-level visual^37^ and somatosensory^38^ discriminations and has been extended to high-level visual processing^39^,^40^,^41^. The present results thus imply that single neurons in the human brain are selective for observed manipulative actions, functioning in much the same manner that MT neurons do in direction discrimination^37^. These action-selective neurons are, most likely, located within the action observation network^42^ for manipulative actions^24^, the parietal and premotor stages of which overlap with the putative human mirror system.

### Action observation depends little on viewpoint

The results of experiment 3 indicate that the discrimination of observed actions is largely independent of viewpoint. While training was more intensive for the frontal viewpoint, the difference between viewpoints vanished once subjects had been tested for both viewpoints. Our results seem to contradict those of de la Rosa *et al*.^43^ who concluded that perception of social interactions were viewpoint-dependent. However, all testing in that study was performed in a single session, thus their results seem to echo our observations during training sessions. Other notable differences in the procedures of their experiment included the use of stick figures derived from 3D motion-capture data and a detecting task in which target actions had to be distinguished from distractor actions. While further studies using additional viewpoints and actions will be required, our initial results clearly suggest that action perception is for the most part viewpoint-independent. Hence, the perception of observed actions is rather different from object perception, which is strongly viewpoint dependent, at least partially due to the self-occlusion of 3D objects^14^,^44^, and from scene perception, which also depends on viewpoint^45^.

The present results are consistent with the recent imaging results of Ferri *et al*.^46^. These authors investigated the effects of stereopsis and viewpoint on the activation elicited by observation of manipulative actions. They found that the main action observation network, including occipito-temporal cortex, phAIP and premotor cortex, was invariant for viewpoint and stereopsis. This network may underlie the discrimination performance once subjects have become familiarized with both viewpoints. Stereopsis in the frontal view however activated a specific network involving left premotor gyrus, left DIPSM and left retro-insular cortex. One may speculate that failure to activate this latter network may have contributed to the difficulty in training subjects in the discrimination for the frontal viewpoint. Our findings are also consistent with the results of Caggiano *et al*.^47^, reporting that premotor mirror neurons are either invariant for viewpoint or are specific for the three main viewpoints tested (egocentric and allocentric frontal and lateral). Hence, this neuronal population could support discrimination for all viewpoints equally well.

### Action observation requires some time

Experiment 6 indicates that relatively short segments after video onset sufficed for subjects to discriminate between grasping and dragging, despite extensive randomization of potentially confounding factors such as hand posture, orientation or position. The 75% threshold, 138 ms, is even shorter than that reported by Tucciarelli *et al*.^11^ for discriminating between grasping and pointing, which we estimate from their Fig. 3 to be 300 ms. There are, however, many differences between the two experiments: 1) the action pairs: grasping-pointing in their study and grasping-dragging in ours, 2) the presence of a mask restricting processing after the end of the stimulus^48^,^49^ in our experiment but not theirs, and 3) viewpoint: egocentric in their study and allocentric (lateral) in ours. The most important factor however maybe the presence of a reach component in their videos described as center-out actions, which contains little information about the later phase of the action (grasping or pointing). These relatively short video segments (about 10 frames) are nonetheless long compared to those required for object and scene perception. Using similar brief stimulus exposure, followed by mask paradigms, Bacon-Mace *et al*.^50^ reported 30 ms to reach 75% correct in distinguishing an animal from a non-animal, Kovacs^49^ *et al*. 30 ms to reach 75% correct in shape discrimination, Green and Oliva^51^ 50 ms to reach 75% threshold in basic scene categorization and Sharan *et al*.^52^ 40 ms to reach 80% correct in material categorization. It is noteworthy that while observed action discrimination requires longer time, it still reached near maximum performance (90% correct) within the duration of a single fixation, which averages 300 ms^53^.

The relatively short timeframe required to discriminate observed actions seems to conflict with the long response times obtained in experiments 1–3. However, the threshold duration is likely to approximate the time required for the sensory processing of the stimulus, which is included in the residual-time parameter of the diffusion model. The latter, which includes both sensory and motor processing, ranged from 0.37 s to 1.1 s in experiments 1–3, leaving sufficient time for the motor execution also included in the residual time. In both experiments the decision time, estimated from the difference between the response time at 100% SL and the residual time, was close to 0.5 s. This is longer than what can be estimated (about 100 ms) for direction discrimination from Palmer *et al*.^30^. In light of the monkey results^37^, it is likely that the decision time in Palmer *et al*.^30^ reflects the integration of signals provided by MT neurons, which fire vigorously to moving noise fields. Hence, the longer decision time in the present study may reflect integration of weaker signals provided by the observed-action selective neurons.

### Action observation is a distinct visual process

We have further demonstrated that action observation cannot be explained by the perception of either the static or the motion components of the action alone. The use of static body frames as a proxy for videos in studying action observation is generally justified by implied motion which has been reported to activate the human motion area MT+ 54,55. However, the recent study^56^ combining MT single-cell recordings and fMRI responses from MT+ indicate that prior human imaging studies demonstrating implied-motion processing in area MT+ can be best explained by sensitivity for low-level features, such as orientation and size, rather than sensitivity for motion implied by animate figures. Our results clearly show that action perception cannot be reduced to presentations of static frames from a video, even if these are technically more convenient^57^,^58^. Indeed, we found that thresholds for observed action discrimination were systematically lower for videos than for static frames, irrespective of how theses static frames were chosen. Extrapolating to 100% signal, performance for static frames rarely reached 100% correct, unlike the performance for videos. Our results are consistent with a large body of action-observation imaging studies, in which static frames and local motion stimuli were used as control stimuli to map regions sensitive to observed actions^25^,^59^.

On the other hand, performance for static frames was clearly higher than for local motion. The reasons may be twofold. First, the main cue for extracting action from the retinal input is the deformation of the body or body part^60^,^61^. This information is extracted from the sequence of body snapshots by so-called snapshot neurons in the STS which respond as strongly, on average, to static frames as they do to the sequence of frames, as their name suggests. The snapshot selectivity, however, explained only 32% of the action selectivity indicating the importance of the sequence of snapshots^60^. Second, responses of so-called STS motion neurons, i.e. action-selective neurons that on average respond only weakly to static frames, correlate poorly with instantaneous speed of a body part, with the speed history most likely determining their selectivity^60^. In principle, motion neurons may also react to more complex flow patterns, but this is less likely, insofar as these neurons generally responded to greatly reduced action configurations, including the single moving dot corresponding to the wrist. Thus, static frames contain partial information about observed actions and action selective neurons are able to exploit this information by being history or sequence sensitive. The partial information contained in static frames, however, can also be exploited by subjects compelled to make decisions about the corresponding actions. The differences between the two action-pairs in experiment 5 indicate that the amount of partial information included in the static frames may depend on the action, in that certain hand shapes might be more diagnostic than others, again consistent with the single cell results of Vangeneugden *et al*.^60^.

## Conclusions

Observed action perception differs in nature from object and scene perception as it implies integration of visual information from several subsequent frames. This distinction in nature explains the longer stimulus duration required for OAP compared to scene and object perception, for which even a single frame^62^ can suffice. Since this sequence of frames captures the body (part) movements, bringing other sides of the body (part) into view, it also explains why OAP is less susceptible to self-occlusion, and thus less viewpoint dependent, than is object and scene perception.

While observed-action perception differs in important ways from other aspects of high level vision, observed action discrimination can be modelled as a diffusion process, indicating that differences from the other aspects of high-level vision is mainly due to the sensory process accruing evidence, and are not attributable to the decision stage which can be similar and possibly shared with these other aspects of high-level vision^22^. Although further work is needed to explore the generality of our results by examining action classes other than manipulation, they imply that observed manipulative actions are processed by neurons selective for such actions. Studies to locate these neurons are currently under way.

## Additional Information

**How to cite this article**: Platonov, A. and Orban, G. A. Action observation: the less-explored part of higher-order vision. *Sci. Rep.* **6**, 36742; doi: 10.1038/srep36742 (2016).

**Publisher’s note:** Springer Nature remains neutral with regard to jurisdictional claims in published maps and institutional affiliations.

## Supplementary Material

Supplementary Information

## Figures and Tables

**Table 1:** Overview of experiments 1–6.

Experiment	Tested actions	PD	SL	Subjects
1	action pair 1	2^o^	0, 10, 20, 30, 50 and 70%	S1–S5
		13^o^	0, 6.25, 12.5, 20, 25 and 50%	S6–S9
2	action pair 2	13^o^	0, 6.25, 12.5, 20, 25 and 50%	S2, S3, S6 and S9
3	action pair 1	13^o^	0, 6.25, 12.5, 20, 25 and 50%	S10-S21
4	action pair 1	2^o^	0, 10, 20, 30, 50 and 70%	S1–S3 and S22
5	action pair 1 and 2	13^o^	0, 6.25, 12.5, 25 and 50%	S2, S3, S9 and S22
6	action pair 2	—	100%	S23–S32

**Table 2:** Parameter values calculated for proportional-rate diffusion model, fitting the results of experiments 1 and 2 (A’ = normalized bound; k = sensitivity; t_R_ = mean residual time in s), threshold ratio, estimated 75% accuracy threshold, and quality of fit (L = likelihood).

	Subject	*A’*	*k*	*t*_*R*_	Threshold ratio	Threshold (75%)	ln(L)
Exp 1	S1	1.53	28.7	0.30	3.50	12.5	23.6
	S2	1.45	27.1	0.33	3.49	14.0	15.8
	S3	1.22	37.8	1.14	3.50	11.9	12.1
	S4	1.26	31.6	0.76	3.49	13.8	15.4
	S5	0.78	49.1	1.06	3.49	14.4	14.8
	S6	1.38	16.9	0.13	3.49	23.7	5.71
	S7	0.93	36.0	0.76	3.48	16.5	9.96
	S8	1.51	19.5	0.04	3.49	18.7	5.89
	S9	1.25	21.1	0.43	3.49	20.9	24.8
	Mean (SD)	1.11 (0.30)	29.8 (10.2)	0.55 (0.40)	3.49 (0.01)	16.3 (4.04)	
Exp 2	S2	1.04	45.2	1.50	3.48	11.7	10.2
	S3	1.31	40.2	1.23	3.50	10.4	32.5
	S6	0.73	69.0	1.40	3.50	10.9	11.4
	S9	1.38	60.7	1.11	3.47	6.6	14.1
	Mean (SD)	1.26 (0.24)	53.8 (13.4)	1.31 (0.17)	3.49 (0.02)	9.9 (2.26)	

**Table 3:** Experiment 3.

	Subject	IT	MT	FT	VS
Group 1	S10	40.5	43.8	36.6	31.6
	S11	47.0	34.2	27.8	23.1
	S12	42.0	34.3	36.3	34.3
	S13	19.8	22.7	18.4	14.6
	S14	34.6	39.8	24.5	21.1
	S15	33.5	25.5	19.3	16.9
	Mean ± SD	36.2 ± 9.46	33.4 ± 8.10	27.2 ± 7.98	23.6 ± 7.89
Group 2	S16	42.4	24.3	20.5	26.7
	S17	24.8	22.1	20.6	26.9
	S18	18.8	28.3	18.8	23.7
	S19	21.6	27.5	20.9	22.5
	S20	18.3	30.1	18.2	22.7
	S21	24.2	23.7	19.2	17.2
	Mean ± SD	25.0 ± 8.93	26 ± 3.09	19.7 ± 1.11	23.3 ± 3.55

Accuracy thresholds in the initial training session (IT), middle training session (MT), final training session (FT) and after a viewpoint switch (VS) for group 1 & 2 subjects.

Two-way ANOVA of IT, MT and FT thresholds: Main effect Session (IT, MT, FT): (F2, 35 = 3.57, p < 0.05); Main effect: Group (1, 2). (F1, 35 = 13.7, p < 0.01); Interaction. (F2, 35 = 0.28, p > 0.75).

**Table 4:** Experiment 3.

	Subject	MT	FT	VS
Group 1	S10	152.7	127.7	110.2
	S11	119.3	96.8	80.5
	S12	119.6	126.7	119.5
	S13	79.3	64.1	50.9
	S14	138.7	85.3	73.7
	S15	89.1	67.1	58.9
	Mean ± SD	116.5 ± 28.1	94.6 ± 27.9	82.3 ± 27.5
Group 2	S16	84.8	71.6	93.1
	S17	77.1	71.7	93.7
	S18	98.6	65.5	82.7
	S19	95.8	72.9	78.3
	S20	104.9	63.6	79.3
	S21	82.7	66.8	60.0
	Mean ± SD	90.7 ± 10.7	68.7 ± 3.87	81.2 ± 12.3

Half-way response time thresholds in middle training session (MT), final training session (FT) and after a viewpoint switch (VS) for group 1 & 2 subjects.
