# Supplementary material for: Action observation: the less-explored part of higher-order vision
Source: Sci Rep. 2016 Nov 18;6:36742. doi: 10.1038/srep36742 (PMC5114682; doi:10.1038/srep36742)
Supplement: Supplementary Information [file srep36742-s1.pdf]

Supplementary Information:

Action Observation: the less-explored part of higher-order vision

Artem Platonov and Guy A Orban*

Supplementary text:

Training procedure

Before participating in experiments 1-5, all observers received equal training in discriminating between the rolling and rotating actions. In the beginning, observers had to complete a familiarization block which contained 30 video clips at 100% signal level chosen pseudo-randomly such that a block included 15 (out of 40) versions of the video clips per action exemplar. Observers responded with a button press at the end of a trial and received an auditory feedback with a low-pitched beep-tone indicating a correct response and a high-pitched tone indicating an incorrect response. After subjects learned which button corresponded to which action, they were presented with another familiarization block with feedback to confirm that they could perform the task. If it appeared that the number of errors exceeded 10%, subjects had to perform another familiarization block. The procedure was repeated until the number of errors was 10% or less. If after 5 familiarization blocks, subjects were still unable to perform the task correctly they did not participate in the experiment.

After completing the familiarization, the subjects were given six 240-trial training blocks (presented in 3 sessions), in which the signal level was manipulated as in the experimental blocks. In addition, each training block was preceded by a familiarization block in which videos without noise were presented. Subjects were given instructions to respond either at the end of the video in the first two training blocks, or as soon as they knew the answer, in the third and subsequent blocks. After finishing training, subjects started with the actual experiment in the next session.

Supplementary table 1: *Gaze precision measured by standard deviation (SD) of horizontal and vertical eye positions and percent rejected trials for all experiments.*

Experiment	Subject	Eye Position SD		% Rejected trials
		Horizontal	Vertical	
1	S1	2.38	2.17	58
1	S2	1.05	0.89	53
1	S3	1.25	0.94	5
1	S4	0.69	0.48	4
1	S5	0.59	0.60	2
1	S6	1.45	0.58	7
1	S7	0.82	0.75	4
1	S8	0.90	0.37	2
1	S9	0.89	0.52	5
2	S2	1.04	0.76	57
2	S3	1.17	0.65	9
2	S6	1.00	0.81	14
2	S9	0.98	0.55	11
3 (FT)	S10	1.31	1.18	4
3 (FT)	S11	1.07	0.86	9
3 (FT)	S12	0.99	0.76	3
3 (FT)	S13	1.15	1.02	5
3 (FT)	S14	1.12	0.95	14
3 (FT)	S15	1.04	1.03	15
3 (FT)	S16	1.62	1.04	27
3 (FT)	S17	0.87	0.82	8
3 (FT)	S18	0.94	0.39	2
3 (FT)	S19	0.96	0.57	8
3 (FT)	S20	0.77	0.65	6
3 (FT)	S21	1.19	0.82	6
3 (PT)	S10	1.38	1.40	11
3 (PT)	S11	1.07	0.86	9
3 (PT)	S12	1.17	0.70	17
3 (PT)	S13	1.32	0.75	24
3 (PT)	S14	1.11	1.03	5
3 (PT)	S15	1.24	1.17	8
3 (PT)	S16	1.58	1.33	19

3 (PT)	S17	0.84	0.45	1
3 (PT)	S18	0.83	0.30	0
3 (PT)	S19	0.89	0.52	3
3 (PT)	S20	0.67	0.79	6
3 (PT)	S21	1.25	0.99	4
3 (VS)	S10	1.13	1.11	7
3 (VS)	S11	1.26	0.90	10
3 (VS)	S12	1.22	0.92	8
3 (VS)	S13	1.16	1.36	40
3 (VS)	S14	1.09	0.62	11
3 (VS)	S15	1.06	0.86	11
3 (VS)	S16	1.63	1.24	24
3 (VS)	S17	0.96	0.50	3
3 (VS)	S18	0.74	0.30	2
3 (VS)	S19	0.91	0.56	3
3 (VS)	S20	0.67	0.51	10
3 (VS)	S21	1.05	0.55	1
4	S1	0.55	0.33	0
4	S2	1.06	1.12	58
4	S3	0.86	1.20	22
4	S22	0.62	0.93	18
5	S2	1.04	0.98	3
5	S22	0.97	0.86	12
5	S3	1.16	1.05	21
5	S9	0.67	0.65	2
6 (240)	S23	1.00	0.60	15
6 (240)	S24	1.48	0.99	45
6 (240)	S25	0.81	0.33	8
6 (240)	S26	1.03	1.16	14
6 (240)	S27	3.31	2.49	40
6 (240)	S28	1.04	0.98	27
6 (240)	S29	0.77	0.93	31
6 (240)	S30	1.25	0.38	23
6 (240)	S31	0.62	0.66	17
6 (240)	S32	1.11	0.67	14
6 (420)	S23	0.96	0.84	34

6 (420)	S24	1.39	1.05	44
6 (420)	S25	0.78	0.42	16
6 (420)	S26	1.13	1.04	13
6 (420)	S27	2.45	2.16	43
6 (420)	S28	1.55	0.95	25
6 (420)	S29	1.28	0.68	37
6 (420)	S30	1.26	0.65	12
6 (420)	S31	0.57	0.38	13
6 (420)	S32	0.98	0.71	14
		1.11±0.42	0.85±0.40	

Supplementary Table 2*: Parameter values calculated for proportional-rate diffusion model, fitting the results from sessions in experiment 3 (A’ = normalized bound; k = sensitivity;* t_R *= mean residual time in s), and quality of fit (L =likelihood) for group 1 subjects trained in frontal viewpoint and tested in lateral viewpoint, and group 2 subjects trained in lateral viewpoint and tested in frontal viewpoint.*

Group	Subject	IT				MT				VS			
		A’	***k***	t_R	**ln(L)**	***A’***	***k***	t_R	**ln(L)**	***A’***	***k***	t_R	**ln(L)**
1	S10	1.65	7.62	0.45	16.0	1.18	12.7	0.75	19.8	1.62	10.7	0.17	44.2
	S11	1.76	9.14	0.32	22.3	2.03	7.48	1.07	21.3	1.92	8.49	0.49	18.5
	S12	1.83	8.81	0.79	31.5	1.95	10.1	0.70	10.4	1.07	22.3	0.78	12.7
	S13	2.68	9.04	2.26	47.0	2.03	14.7	0.84	22.2	1.74	21.6	0.01	6.22
	S14	0.06	23.4	1.35	5.81	1.11	20.3	1.46	18.9	1.89	13.7	0.38	35.5
	S15	1.98	10.8	0.56	23.9	1.21	23.6	0.84	7.59	1.41	23.1	0.40	18.3
	Mean (SD)	1.66 (0.87)	11.5 (5.93)	0.96 (0.73)		1.59 (0.46)	14.8 (6.12)	0.94 (0.28)		1.61 (0.32)	16.7 (6.46)	0.37 (0.27)	
2	S16	0.71	31.9	1.22	25.0	1.71	15.7	0.15	18.2	1.70	12.1	0.01	17.9
	S17	1.37	18.2	0.16	10.0	2.34	11.4	1.45	26.3	2.32	8.88	1.68	39.9
	S18	0.85	22.8	1.17	35.6	1.63	18.0	0.41	39.6	1.58	14.6	0.56	14.3
	S19	1.76	11.4	0.01	43.7	1.25	21.1	0.44	24.8	1.77	13.8	0.19	39.3
	S20	0.09	20.9	1.73	20.3	2.10	14.4	0.64	30.5	2.68	9.10	2.26	47.0
	S21	1.00	23.1	0.92	29.9	2.10	13.6	0.89	48.2	1.84	17.3	0.14	29.3
	Mean (SD)	0.96 (0.57)	21.4 (6.72)	0.87 (0.66)		1.86 (0.40)	15.7 (3.44)	0.66 (0.46)		1.98 (0.43)	12.6 (3.28)	0.81 (0.94)	

***Supplementary table 3**: Experiment 3. Threshold ratio in middle training session (MT), final training session (FT) and after a viewpoint switch (VS) for group 1 subjects trained in frontal viewpoint and tested in lateral viewpoint, and group 2 subjects trained in lateral viewpoint and tested in frontal viewpoint.*

	**Subject**	**Threshold ratio**		
		**MT**	**FT**	**VS**
**Group 1**	S10	3.49	3.49	3.49
	S11	3.49	3.48	3.48
	S12	3.49	3.49	3.48
	S13	3.49	3.48	3.49
	S14	3.48	3.48	3.49
	S15	3.49	3.48	3.49
	Mean±SD	3.49±0.004	3.48±0.005	3.49±0.003
**Group 2**	S16	3.49	3.49	3.49
	S17	3.49	3.48	3.48
	S18	3.48	3.48	3.489
	S19	3.48	3.49	3.48
	S20	3.49	3.49	3.49
	S21	3.49	3.48	3.49
	Mean±SD	3.49±0.003	3.49±0.006	3.49±0.005

***Supplementary table 4:** 75% accuracy thresholds, in experiment 4, expressed as percent signal in the full action and static conditions. Values were obtained by averaging the thresholds for rotation and rolling action discrimination.*

Subject	75%-accuracy threshold (action condition)	ln(L)	75%-accuracy threshold (static condition)	ln(L)
S1	8.65	35.2	37.9	33.5
S2	13.3	54.5	88.5	31.8
S3	17.9	42.2	64.1	11.6
S22	24.7	29.6	64.3	36.8
Mean±SD	16.1±6.86		63.7±20.7	

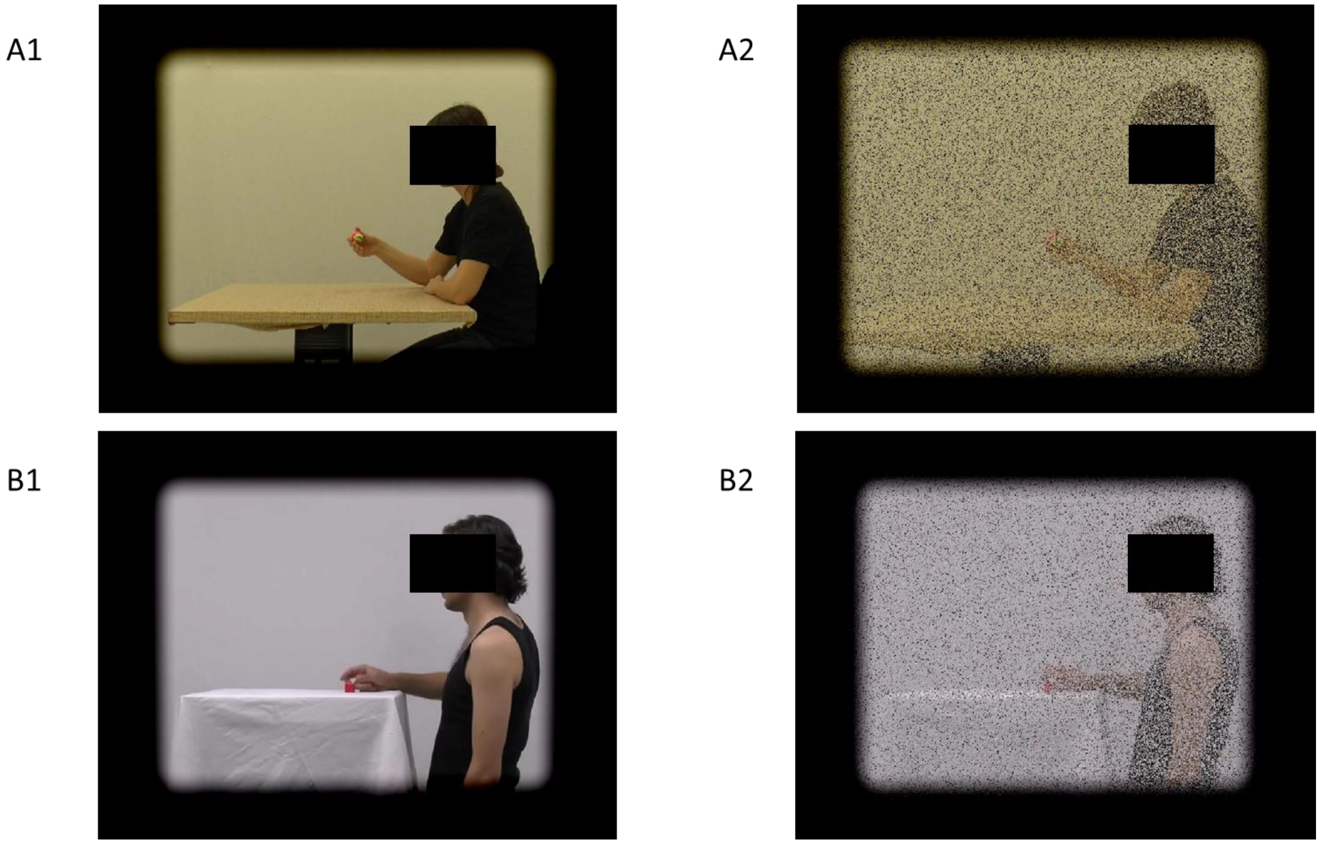

Supplementary Figure 1: *Static frames of Rolling/Rotating (A) and Dragging/Grasping (B) pair with 100% SL (1) and 50% SL (2). The faces of the actors are blacked out here to protect their privacy, but were visible to the subjects.*

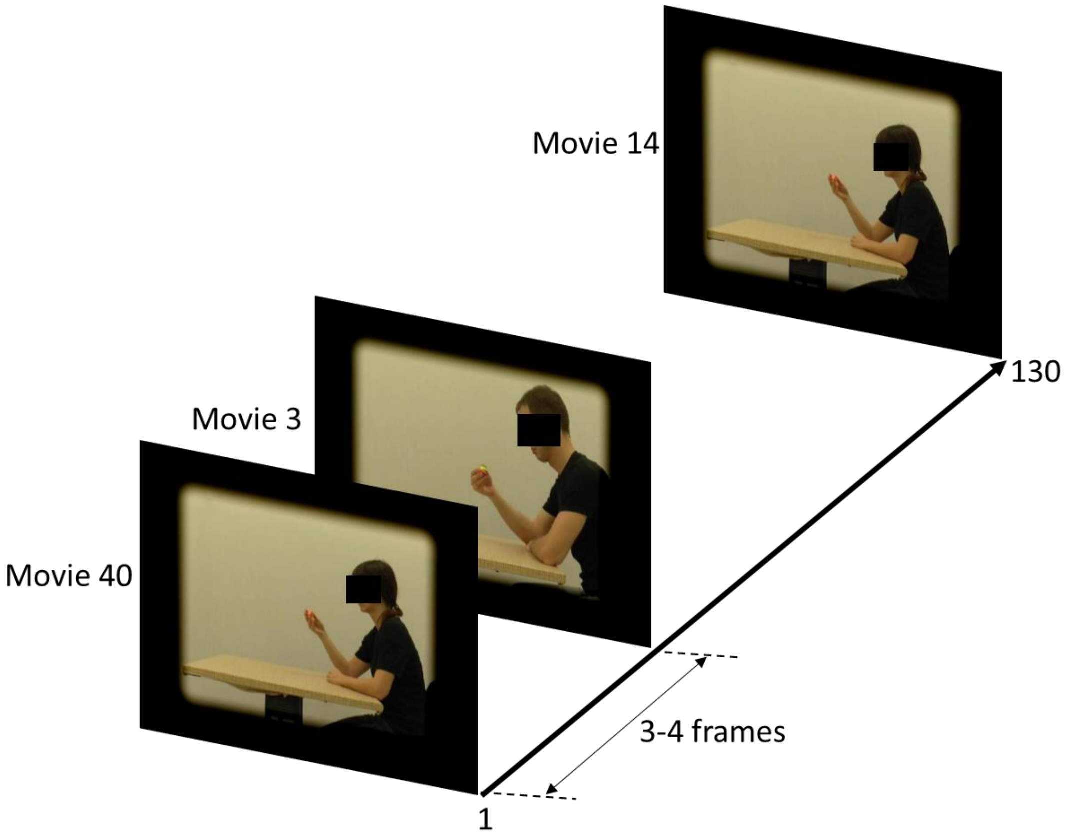

Supplementary Figure 2: *The static stimuli were created by defining 40 frames, uniformly distributed across the video clip length (130 frames), and randomly assigning to them one of the 40 action versions. The faces of the actors are blacked out to protect their privacy, but were visible to the subjects.*

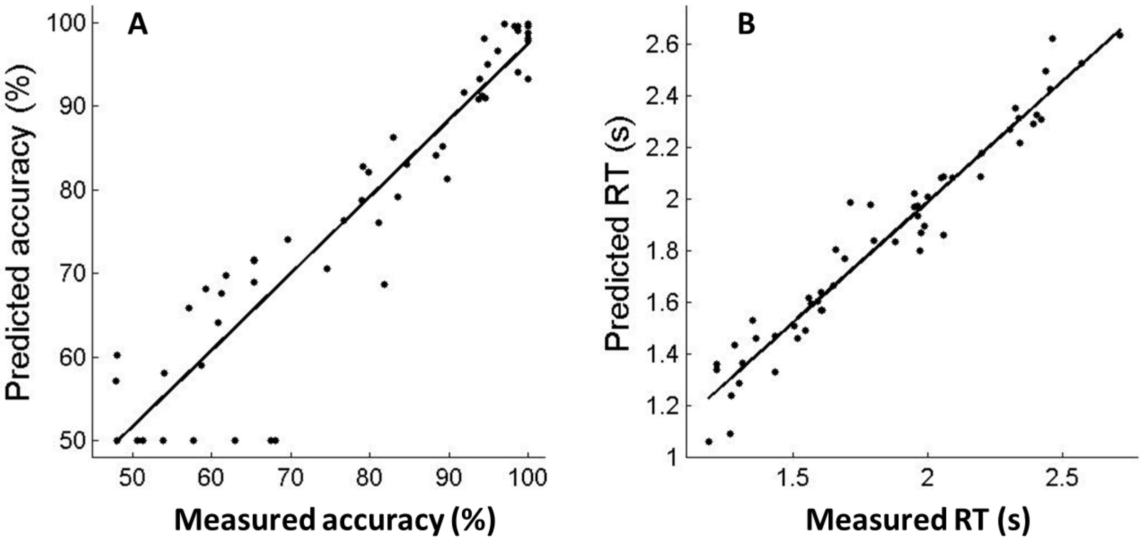

Supplementary Figure 3: *Scatterplot of predicted versus measured accuracy (A) and response time (B), obtained in the experiment 1. Plot shows that both data were well-described by proportion-rate diffusion model (Main text). Dots identify the results from individual subjects.*

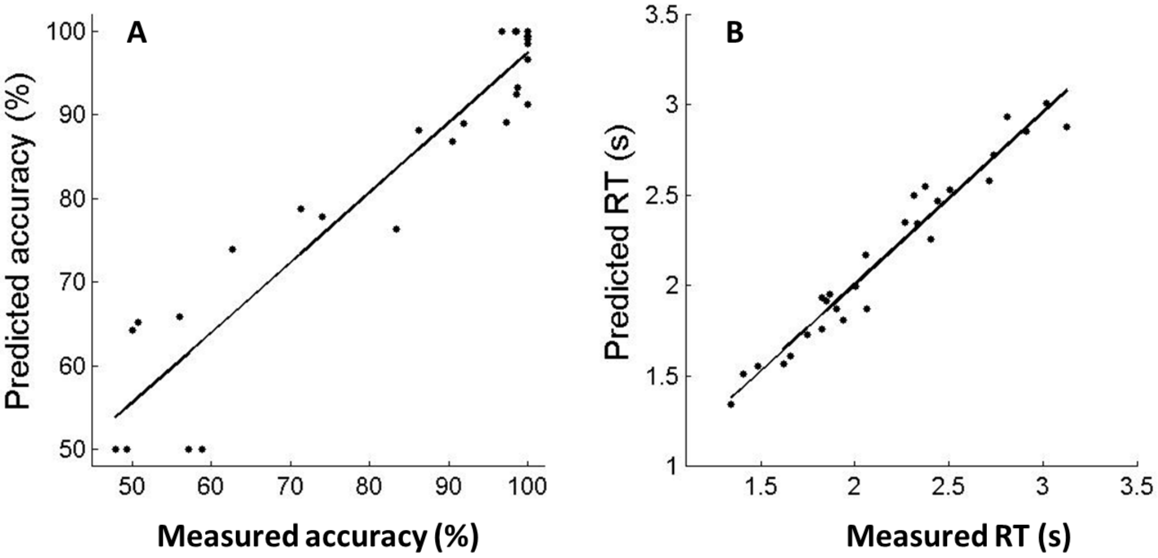

Supplementary Figure 4: *Scatterplot of predicted versus measured accuracy (A) and response time (B), obtained in the experiment 2. Dots identify the results from individual subjects.*

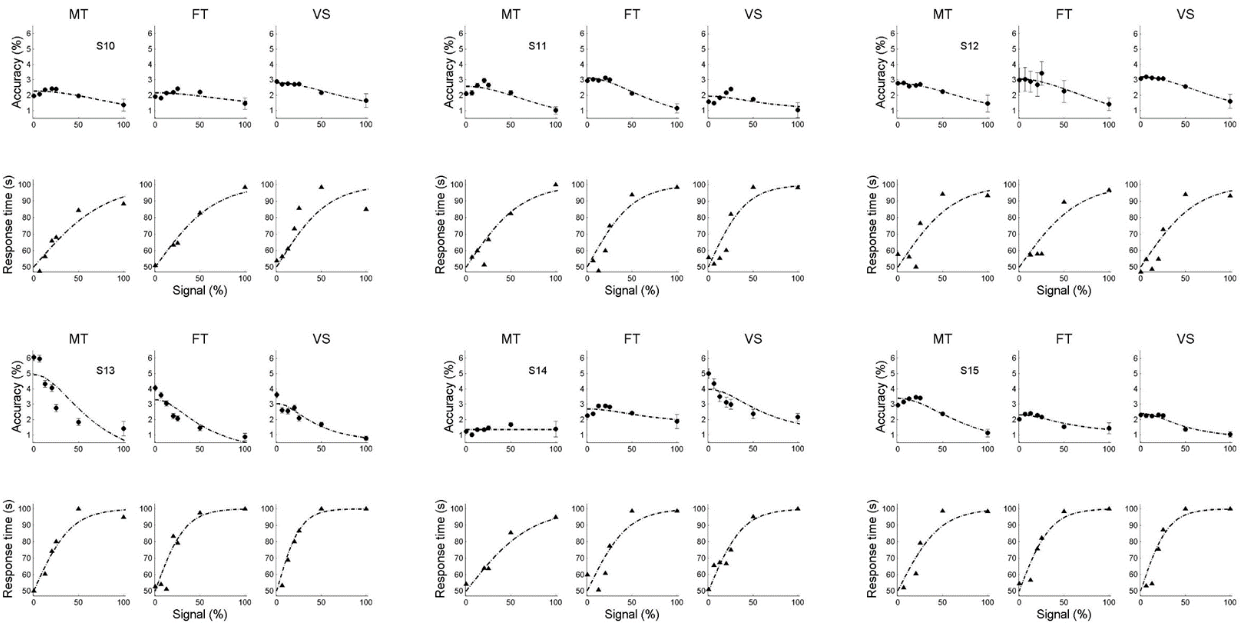

Supplementary Figure 5*: Experiment 3. Individual-subject response times (circles) and accuracy (triangles) plots as a function of signal strength fitted by the proportional-rate diffusion model (lines) for group 1 subjects in MT, FT and VS sessions. Error bars indicate ±1 SEM.*

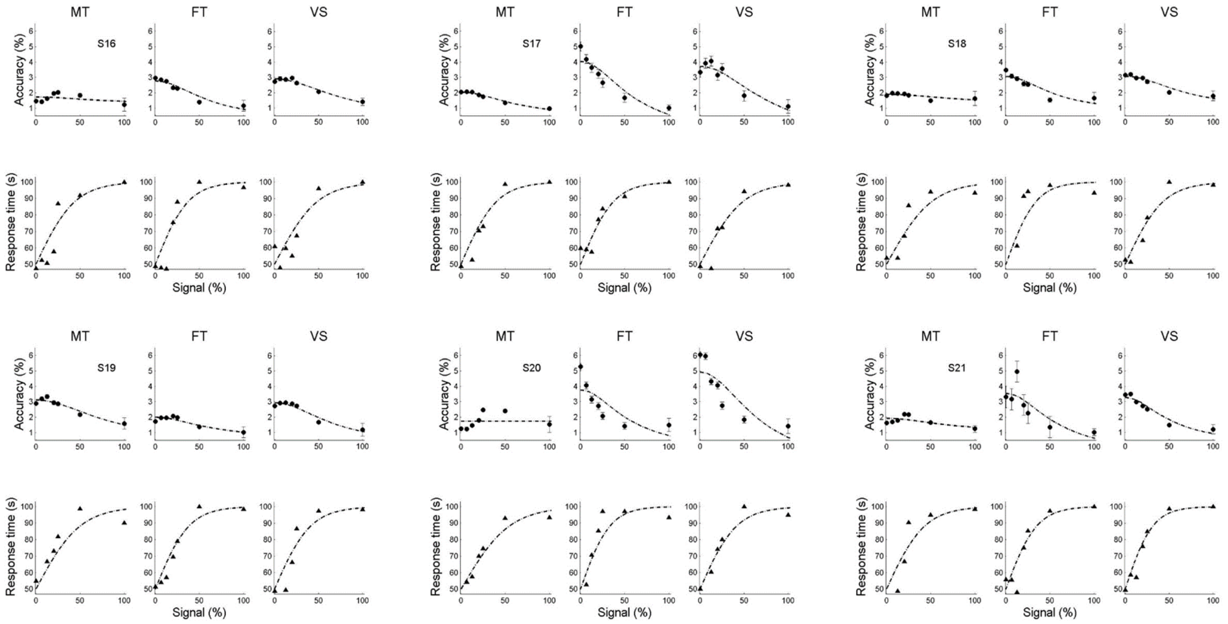

Supplementary Figure 6*: Experiment 3. Individual subject response times (circles) and accuracy (triangles) plots as a function of signal strength fitted by the proportional-rate diffusion model (lines) for group 2 subjects in MT, FT and VS sessions. Error bars indicate ±1 SEM.*

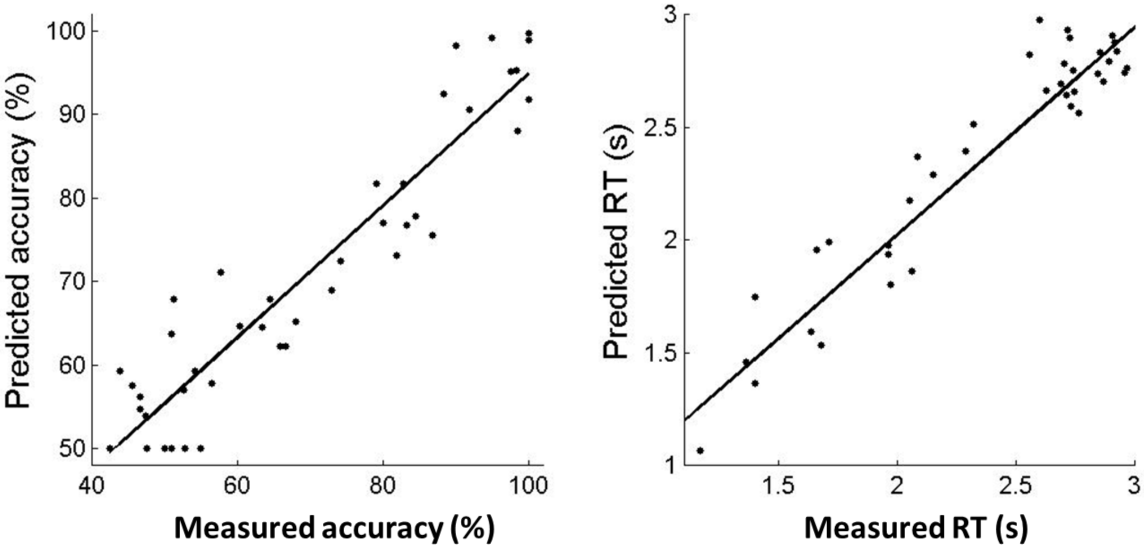

Supplementary Figure 7: *Scatter plot of predicted versus measured accuracy (A) and response time (B), obtained in the MT session of experiment 3. Dots identify the results from individual subjects.*

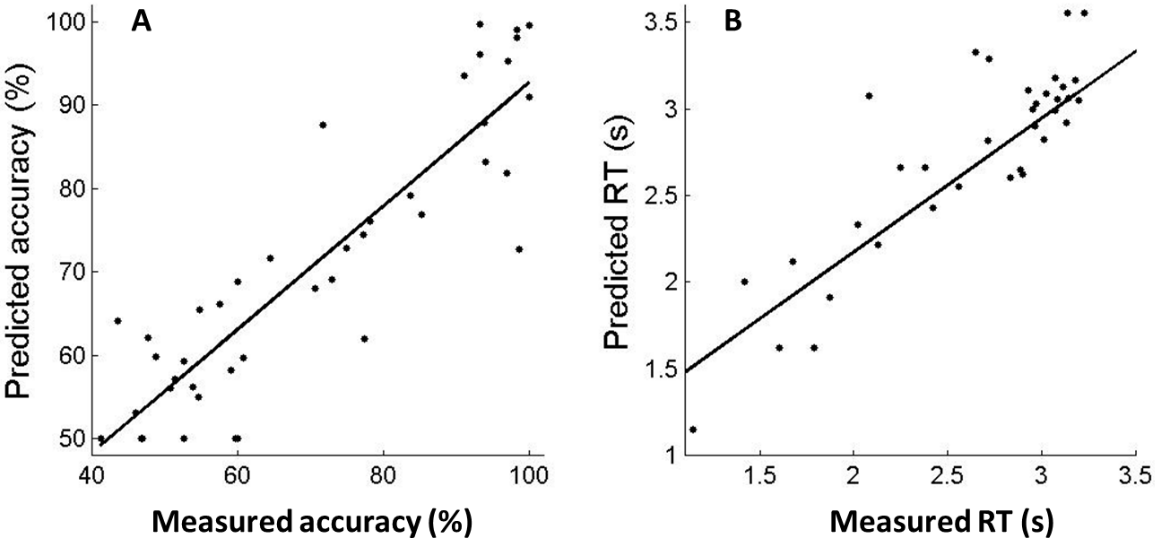

Supplementary Figure 8: *Scatterplot of predicted versus measured accuracy (A) and response time (B), obtained in the FT session in experiment 3. Dots identify the results from individual subjects.*

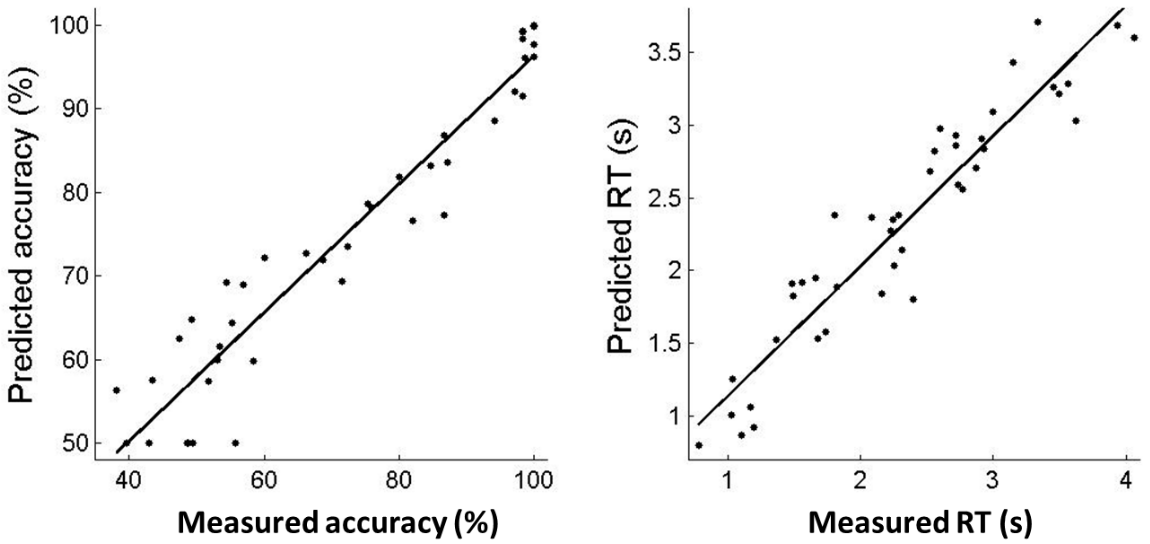

Supplementary Figure 9: *Scatter plot of predicted versus measured accuracy (A) and response time (B), obtained in the VS session in experiment 3. Dots identify the results from individual subjects.*
